# Challenges and opportunities for implementation of substance misuse interventions in conflict-affected populations

**DOI:** 10.1186/s12954-018-0267-1

**Published:** 2018-11-28

**Authors:** M. Claire Greene, Jeremy C. Kane, Kaveh Khoshnood, Peter Ventevogel, Wietse A. Tol

**Affiliations:** 1 Department of Mental Health, Johns Hopkins Bloomberg School of Public Health, 624 North Broadway, Rm. 888, Baltimore, MD 21205 USA; 2 Department of Epidemiology of Microbial Diseases, Yale School of Public Health, New Haven, CT USA; 3 Public Health Section, United Nations High Commissioner for Refugees, Geneva, Switzerland; 4 Peter C. Alderman Foundation, Kampala, Uganda

**Keywords:** Substance misuse, Alcohol misuse, Conflict, Displacement, Implementation

## Abstract

Alcohol and other drug misuse are significant but neglected public health issues in conflict-affected populations. In this article, we review the literature on the challenges and strategies for implementing substance misuse treatment and prevention services in conflict and post-conflict settings in low- and middle-income countries. We identified nine studies describing interventions in conflict-affected populations residing in Afghanistan, Croatia, India, Kenya, Kosovo, Pakistan, and Thailand. Six of these nine studies focused on refugee populations. Reports revealed challenges to intervention implementation, as well as promising practices and recommendations for future implementation that we characterized as existing in the inner and outer contexts of an implementing organization. Challenges existing in the outer context included low political prioritization, lack of coordination and integration, and limited advocacy for access to substance misuse services. Challenges within the inner context related to competing priorities and a shortage of providers. Resource limitations existed in both the inner and outer contexts. Stigma was a challenge that threatened implementation and utilization of substance use services in situations when substance use interventions were not congruent with the roles, structure, values, and authority of the system or implementing organization. Future research should focus on developing, applying, and evaluating strategies for overcoming these challenges in order to make progress toward meeting the need for substance misuse services in conflict-affected populations.

## Burden of substance misuse in conflict and post-conflict settings

Conflict-affected populations experience an elevated risk and burden of alcohol and other drug misuse [1, 2], yet there is substantial variability in patterns of use between cultures and contexts [3]. Substance misuse in conflict and post-conflict settings can exacerbate the concurrent health and social consequences arising from conflict and stifle the recovery process [4, 5]. The mechanisms by which conflict is believed to increase risk for substance misuse include using substances, particularly alcohol, as a means to cope with increased psychosocial distress and adversity in the aftermath of a humanitarian emergency; changes in access and availability of alcohol and other drugs; displacement; and evolving social norms [6, 7]. Epidemiologic estimates of the burden of substance misuse in conflict-affected populations are not always reliable and difficult to compare due to strong heterogeneity of studies with considerable variability in measurement, study design, sampling methods, and characteristics [2]. Important intervention guidelines for humanitarian assistance such as the IASC Guidelines on Mental Health and Psychosocial Support in Emergency Settings [8] and the Sphere Handbook [9] include recommendations for addressing substance use. In practice, however, alcohol and drug misuse are not or insufficiently addressed in the humanitarian response [10]. An analysis of consultations in health facilities in 90 refugee settings showed that the number of consultations for alcohol or substance use problems was low [11]. There is a clear need for further and more standardized research on patterns of alcohol and other drug misuse in conflict-affected populations as well as the implementation and evaluation of treatment and prevention services in these settings [12].

Currently, knowledge on the effectiveness of interventions is limited with very few studies evaluating substance use outcomes with rigorous methods. However, more evidence is available on the feasibility of introducing and implementing substance misuse interventions in various conflict-affected populations. Synthesizing the evidence on the feasibility of interventions is important in its own right because it may inform the design of future intervention studies and contribute to anticipating and potentially circumventing the identified implementation challenges. Thus, the purpose of this article is to review implementation challenges, strategies, and opportunities for alcohol and other drug misuse treatment and prevention interventions in conflict-affected populations.

## Substance misuse interventions in conflict-affected populations

We conducted a scoping review of the academic and unpublished literature to identify articles describing the implementation or evaluation of substance misuse interventions in conflict-affected populations. Academic databases included AnthroSource, Embase, PsycINFO, Published International Literature on Traumatic Stress (PILOTS), PubMed/MEDLINE, and SCOPUS. We also searched five gray literature databases (ALNAP, IRIN, mhpss.net, ReliefWeb, and ACAPS) and four relevant United Nations agency websites (UNHCR, WHO, UNODC, and UNICEF) and conducted targeted searches of *Intervention: the Journal of Mental Health and Psychosocial Support in Conflict Affected Areas*. All searches included key terms focused on substance misuse (e.g., “drug,” “alcohol,” “substance”), interventions (e.g., “intervention,” “treatment,” “therapy,” “program”), and refugees or conflict-affected populations (e.g., “refugee,” “war,” “conflict,” “post-conflict,” “displaced”). Searches were initially conducted in April 2016 and updated in October 2018. In the updated searches, we added a search concept that included a list of conflict and post-conflict countries using data from the United Nations Peacekeeping database [13]. In total, we screened 12,994 articles for potential relevance to this review. We included all articles that were published in English and described a prevention or treatment intervention for refugees or conflict-affected persons in low- and middle-income countries. Factors relating to implementation were extracted from the included studies by two authors (MCG, JCK). We clustered the challenges and strategies for implementation into themes that emerged during the article review and data extraction process and compared them with existing implementation science frameworks that could be used to synthesize and present these findings.

We identified six studies conducted in refugee populations [4, 14–20] and three studies conducted in other conflict-affected populations [21–24]. As shown in Table 1, one study conducted in Kosovo focused on strengthening collective resilience to develop systems and capacity to address substance misuse in the community following a dramatic increase in the prevalence of substance use and disorder after the war. The Linking Human Systems (LINC) family- and community-based community resilience intervention relies on respected community members to facilitate communication and access to resources within the community to address priority health and social concerns related to a recent collective traumatic event or loss. In Kosovo, the LINC intervention resulted in the establishment of substance misuse resource centers focused on education and treatment services [22].

**Table 1:** Characteristics of substance misuse interventions in conflict-affected populations

Author, year	Country	Population	Objective (design)	Intervention	Implementation challenges
Abatemarco, 2004; West, 2008	Croatia	Middle school students (49% female, 10–14 years)	Delay alcohol initiation and reduce use (cluster RCT)	School-based educational intervention	Human resources, community buy-in, competing priorities, government turnover
Agani, 2010	Kosovo	Family and community members	Strengthen community resilience and resources to address substance misuse (no evaluation)	Linking Human Systems Community Resilience Model (LINC)	None reported
Armstrong, 2010; Kumar, 2009	India	People who inject drugs; mean age, 30–31 years; mostly male	Reduce opioid use and related harm (Armstrong, 2010: prospective; Kumar, 2009: cross-sectional)	Sublingual buprenorphine	Sustainable funding
Bolton, 2014	Thailand	Adult Burmese refugees	Reduce alcohol and co-occurring mental health problems (RCT)	Transdiagnostic psychotherapy including brief motivational interviewing	Supervision, communication
DARE, 2014; Lai, 2014	Thailand	Refugees (male and female, adult and adolescent)	Treat and prevent substance use disorder (pre-, post-test)	Drug and Alcohol Recovery Education (DARE) and rehabilitation	None reported
Ezard, 2010	Thailand	Adult male Burmese refugees	Detect hazardous alcohol use and motivate reduction in risky drinking (cross-sectional)	Screening and brief intervention	Stigma, mistrust
Todd, 2009; Todd, 2015	Afghanistan	Males who inject drugs; 95% refugees; mean age, 32 years	Reduce injection-related harms (qualitative)	Harm reduction and needle exchange program	Insufficient services, logistics, organization
UNODC, 2003; UNODC, 2004	Afghanistan, Pakistan refugee camps	Adults (mostly refugees) with substance use problems	Prevent and reduce substance misuse and promote reintegration (no evaluation)	Capacity building, community awareness, rehabilitation	None reported
Widmann, 2017	Kenya	Male (50% refugees); mean age, 27 years	Reduce khat use (RCT)	ASSIST-linked brief intervention	Criminality, political tension

Abbreviations: *ASSIST* Alcohol, Smoking and Substance Involvement Screening Test, *RCT* randomized controlled trial, *UNODC* United Nations Office on Drugs and Crime

We identified one universal prevention, three indicated prevention, and one harm reduction intervention in this scoping review. The universal prevention intervention, Project Northland, is a multi-year, school-based intervention aimed to delay alcohol initiation and reduce drinking among middle school students in Croatia. Using an ecological approach, this intervention engages with the students, their parents, and peers to mitigate risk factors for alcohol initiation and risky drinking [21, 23]. We identified three indicated prevention interventions for refugees in Thailand and Kenya, all of which aimed to reduce hazardous alcohol or khat use through brief motivation-based interventions in camp and urban settings [14–16]. Notably, the focus of these interventions was to reduce risky substance use, as opposed to preventing the incidence of alcohol use disorder, which is the traditional target of indicated prevention approaches [25]. Two of these interventions targeted Burmese refugees living in camps along the Thai-Burma border who displayed risky drinking patterns. The first study screened men in outpatient care settings using the Alcohol Use Disorders Identification Test (AUDIT) and provided brief advice on cutting down alcohol use to men who met criteria for high-risk drinking. These men were also offered individual counseling. Individuals who met criteria for possible alcohol dependence were referred to specialized substance use services [15]. The second brief intervention for Burmese refugees in Thailand integrated screening and brief interventions for hazardous alcohol use into a Common Elements Treatment Approach (CETA), a transdiagnostic psychotherapy that was developed for treatment of depression, anxiety, and post-traumatic stress, among other common mental health problems. All participants were screened for risky drinking using the AUDIT, and those who met the criteria were administered the brief intervention as part of their CETA program [14]. The third brief indicated prevention intervention recruited male Somali khat chewers, about half of whom were refugees, who were motivated to stop or reduce their khat use. Instead of being integrated into another service, the brief motivation-based intervention was administered as a stand-alone single-session service [16]. In addition to these prevention interventions, we identified one qualitative evaluation of the implementation of harm reduction services for people who inject drugs in Afghanistan, most of whom had been refugees in Iran or Pakistan. Although Kabul had four no-cost substance use treatment centers at the time of the study, they had limited capacity and typically there was no availability forcing most treatment-seeking people who inject drugs to utilize community-based harm reduction programs. The primary activity of these harm reduction programs was syringe exchange, but they also included infectious disease prevention, counseling, psychoeducation, and naloxone distribution [20, 26].

The remaining three interventions focused on treatment of alcohol and other drug problems through rehabilitation programs among refugees in Thailand, Afghanistan, and Pakistan and medication-assisted treatment for opioid use disorder in northeast India [17–19, 24]. The DARE program provided inpatient and outpatient services to refugees and migrant workers in Thailand. These services were delivered in four phases: detoxification using Burmese herbal medicines and therapies, recovery counseling focused on self-care and coping, psychoeducation and harm reduction, and reintegration [17]. The remaining intervention was developed by the United Nations Office of Drugs and Crime (UNODC) and focused on providing community-based services for substance use prevention and treatment. Services included home-based detoxification, coordinated aftercare (e.g., self-help groups), and social reintegration (e.g., livelihood training, community volunteering). The programs in Thailand, Afghanistan, and Pakistan also included community awareness and education campaigns to prevent substance use [18, 19]. The combined medication-assisted treatment and harm reduction program in Nagaland and Manipur, India, provided buprenorphine to individuals with opioid use disorder seeking care through non-governmental organization-administered HIV prevention programs. Clients were predominantly males (92%) who had been injecting heroin, Spasmo Proxyvon, or other opioids. Buprenorphine was prescribed by trained health care workers under the supervision of medical doctors working in community-based drop-in centers, which also provided peer support and education, HIV counseling and testing, STI treatment, condom distribution, and primary care [24, 27].

Three of the included studies employed experimental evaluations that allow for inferences to be made about the effectiveness of these interventions [14, 16, 21, 23]. The remaining studies were observational, non-controlled, or focused on describing the intervention without an evaluation of effectiveness. As per the eligibility criteria for this review, all studies included examination of feasibility and process outputs. Therefore, it is not possible to make inferences on effectiveness, but these studies provide important insights into challenges in implementation of these interventions that may inform substance misuse programming and intervention research in the future.

## Challenges and strategies for implementation of substance misuse interventions in conflict-affected populations

**Fig. 1:**
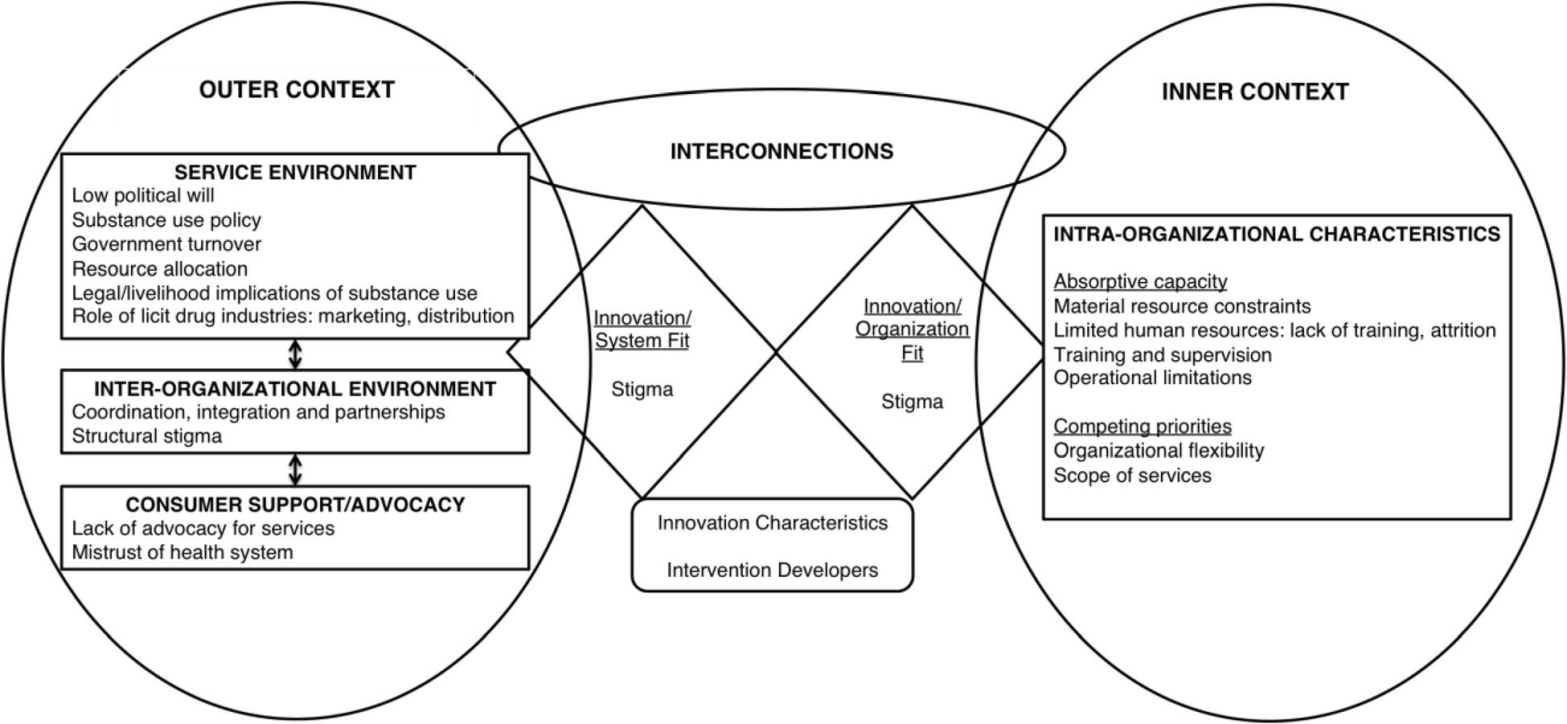
Challenges for implementation of substance use treatment and prevention services in conflict-affected populations. We adapted the Exploration, Preparation, Implementation and Sustainment (EPIS) framework developed by Aarons and colleagues to describe challenges for implementing substance use treatment and prevention interventions in conflict-affected populations into those existing within the inner and outer context [29]. Challenges related to the service environment, inter-organizational environment, and lack of consumer advocacy were identified in the outer context. Inner context implementation challenges relating to characteristics of the program or implementing organization included absorptive capacity and competing priorities. Lack of innovation-values fit of substance use services within the system and organization manifested through multiple forms of stigma

We characterized the challenges and strategies referenced in these articles using Aarons and colleagues’ Exploration, Preparation, Implementation and Sustainment (EPIS) framework. The EPIS framework organizes implementation factors into those existing in the inner or outer context (Fig. 1). The phases at which implementation challenges and strategies may emerge in the inner and/or outer context begin with the moment an organization recognizes an opportunity and explores options to improve service delivery (i.e., exploration phase), followed by the decision to adopt a specific evidence-based strategy (i.e., preparation phase), introducing the new strategy (i.e., implementation phase), and ultimately maintaining the implementation of the new strategy (i.e., sustainment phase). At the intersection of the inner and outer contexts lies the fit of the intervention within the organization and system. More specifically, this refers to the agreement between the roles, structure, values, and authority of the intervention, organization, and system [28, 29]. In contrast to previous substance use interventions that have used this framework to develop an implementation and evaluation strategy [30], we used the EPIS framework to classify implementation challenges that were reported after an intervention had been implemented.

### Outer context

In the outer context, we identified implementation challenges existing across EPIS phases. These challenges related to the service environment, inter-organizational environment, and consumer advocacy and support.

#### Service environment

Barriers pertaining to the service environment were primarily related to the sociopolitical context in post-conflict settings and included low political will to prioritize or provide resources to support substance misuse services as well as the criminalization and stigma toward illicit substance use and misuse. These challenges were often compounded in conflict-affected settings by frequent and unpredictable government turnover, which resulted in changes to resource allocation, regulations, and support for services [21]. One example of the impact that changes in governance can cause is the case of the Russian annexation of Crimea in 2014, which was followed by a ban on opioid agonist treatment by the Russian government, thus leaving people who were on medication-assisted treatment without access to care [31, 32]. Similarly, the possibility of legal or livelihood consequences, particularly in refugee camp settings where policies prohibiting alcohol or other drug use may have implications for access to humanitarian aid (e.g., food rations) or the resettlement process, may introduce barriers to service utilization and disclosure [15]. Another outer context challenge relating to the service environment is the role of other stakeholders, such as the alcohol industry. Qualitative interviews with policymakers, humanitarian aid workers, and other local stakeholders revealed that licit substance industries may capitalize on post-conflict environments where governments may weakly regulate or enforce the marketing and distribution of alcohol or the drugs, which is likely to increase access and use of substances [33, 34].

#### Inter-organizational environment

In general, successful implementation of substance misuse interventions at the organizational level was largely attributed to strong partnerships with community stakeholders and ensuring a sense of ownership over these services such that they became prioritized by and part of the health and education sector’s mandate [15, 23]. In our review, there were several examples of situations where institutional biases toward substance use (i.e., structural stigma) introduced barriers to delivery and utilization of substance use services. Structural stigma may also be a driver of low prioritization, acceptance, or adoption of substance use services by a variety of organizations and stakeholders. For individuals with substance use problems, structural stigma may present in the form of negative attitudes held by representatives of medical and other institutions that exclude these individuals from accessing the same level or quality of care [35]. This form of stigma can exacerbate client’s resistance to seek treatment and reduce access among individuals that are motivated to seek services. For example, public transportation drivers reportedly prohibited people who were known to inject drugs from riding buses or other modes of transportation used to travel to harm reduction centers in Afghanistan. Furthermore, clients who walk to health care facilities reported being harassed by police, which similarly made it difficult to access harm reduction services [20]. To circumvent this challenge, a medication-assisted treatment and harm reduction program in India carried out advocacy with police, militant groups, policymakers, and other stakeholders to ensure that they did not interfere with client’s access to services or program activities [27].

#### Consumer advocacy and support

There is a need for advocacy promoting the provision of substance misuse prevention and treatment services for populations affected by conflict directed toward governments, humanitarian agencies, and policymakers that are responsible for allocating resources and determining health system priorities in the aftermath of a conflict or other emergency. Only one study included in this review described advocacy efforts as part of their implementation strategy but did not specify whether consumers were involved in these activities [27]. Recommended strategies for increasing the visibility of alcohol and other drug misuse as a priority in conflict-affected populations include increasing awareness regarding the harmful effects of substance misuse, sharing information and expertise on the topic, and improving the quality of data documenting substance misuse and related consequences in conflict-affected populations [33].

### Inner context

Barriers residing within the inner context were related to intra-organizational factors, primarily those that dealt with human and material resource capacity as well as competing priorities that affected the scope of services provided within the organization.

#### Absorptive capacity

In our review, we identified factors relating to absorptive capacity that challenged implementation of substance use interventions in conflict-affected populations including human and material resource limitations and limited knowledge of evidence-based interventions. Resource limitations in low- and middle-income contexts are often exacerbated in conflict and post-conflict settings where pre-existing facilities, services, and other resources may become inaccessible or seriously limited, forcing remaining providers to operate within critically resource-constrained circumstances [36, 37]. Additionally, when services are provided within the context of research or humanitarian funding, the sustainability of programming may be threatened when resources are no longer available after external humanitarian agencies leave and the responsibility to support these programs is transferred to other stakeholders (e.g., the government) [24, 27].

Loss of human resources due to attrition of providers and other personnel in humanitarian settings further reduces access to services for people with substance use problems [38]. Lack of specialized providers is a common challenge for mental health programs more broadly in low-income and humanitarian settings [39, 40]. Growing evidence suggests that the capacity of lay health workers to provide mental health, including substance misuse, interventions is sufficient and serves as a cost-effective strategy to alleviate the unmet need for care due to the shortage of specialty health providers in many low-income settings [41]. One included study employed a “training of trainers” model whereby local personnel served as trainers for school teachers, who ultimately delivered a school-based alcohol misuse prevention intervention to youth in a post-conflict setting. Although the teacher trainings were reportedly successful, the study investigators cite the process of identifying and adequately training the trainers to be a challenge [21], and previous researchers have noted the importance of ensuring trainers themselves have sufficient time to practice newly learned skills, before becoming trainers [42].

Building capacity of intervention providers, particularly non-specialized providers without prior training, often requires high levels of monitoring and supervision that may be impractical for long-term, real-world program implementation, particularly when challenged by language and cultural barriers [14, 23]. Effective capacity building for lay providers of psychosocial interventions requires more than one-off didactic trainings and must incorporate ongoing supervision, active learning, and organizational support to ensure fidelity [42]. Challenges in the training and supervision process need to also be addressed through evaluations of provider competency and establishing realistic but adequate supervision mechanisms to ensure fidelity. Deficits in provider competencies can be recognized by clients, which may discourage continued engagement and retention in care [20]. One study quoted that providers were giving clients “empty promises of treatment,” which may reflect insufficient provider competency or lack of resources precluding optimal implementation of substance misuse interventions [20]. Additionally, inconsistent operating hours and schedules, discrimination and harassment, and lack of resources and supplies (e.g., syringes, methadone) limit providers’ ability to deliver care [20]. To address absorptive capacity challenges, it is important that providers are equipped with practical guidelines that may be implemented with fidelity by non-specialists, particularly in settings where there are few or no available specialty substance misuse treatment providers—and that sufficiently long-term capacity building strategies are planned for from the beginning, especially in humanitarian response [43].

#### Competing priorities

Competing priorities and scope of services were another implementation challenge relating to intra-organizational characteristics. For example, clients of a harm reduction program reported that the services they needed or prioritized were not available and recommended that organizations expand their scope of services. Some of these services were specific to substance misuse treatment and harm reduction (e.g., medication-assisted treatment), while others would require integrated or coordinated services across sectors (e.g., shelter, employment, counseling) [20]. In humanitarian settings, the need for inter-sectoral collaboration is critical given the needs of communities who have often lost their assets through disaster or displacement and may require concurrent interventions for basic needs and social problems (e.g. livelihoods, protection, and education), as well as physical and mental health issues [8, 40]. Organizational flexibility to accommodate the increased demand and ensure that access to these services are maintained despite disruptions in distribution of supplies, transportation systems, or destruction of facilities is a critical challenge in acute post-conflict and disaster settings [44].

### System- and organization-level innovation-values fit

Innovation-values fit refers to the degree to which a service is congruent with the values and goals of a system, an organization, its leadership, providers, and the consumer. Stigma and values are largely interconnected constructs and may influence the innovation-values fit of substance use services within a given organization or system. Stigma has been thought to be the manifestation of social devaluation or a means of social control to deter certain behaviors, such as substance use [45]. Across studies, settings, stakeholders, and implementation levels (i.e., inner vs. outer context), stigma was consistently described as challenge for implementation and typically reduced access to services. Stigma, particularly self-stigma, was referenced in numerous studies and cited as a source of resistance to seek care, which limits service utilization and compromises implementation of substance misuse interventions. Self-stigma (i.e., self-devaluation and fear resulting from identification with a stigmatized group), perceived stigma from providers and the community, and mistrust are common reasons that individuals resist seeking treatment for substance use problems [46, 47]. Several studies have reported on the impact of policies prohibiting substance use, particularly those with legal and livelihood consequences, which reinforced structural stigma and resulted in disincentives to disclose substance use to providers and other professionals in conflict-affected and displaced populations [1, 15, 46, 48]. Structural stigma, which was also considered a contributor to inter-organizational implementation challenges, may reinforce self-stigma and provider stigma. Stigma raises critical ethical concerns, which should impact the delivery of substance use services. For example, when implementing substance use assessment and interventions, particularly in situations where substance use is criminalized or have implications for the receipt of other services, the provider’s knowledge is not highly protected or there is a lack of privacy; additional protections should be introduced to ensure the safety of clients and improve utilization of substance use interventions.

## Discussion

Availability and access to services for substance use disorders are limited for conflict-affected populations. This review identified multiple implementation challenges for such services existing within service delivery organizations as well as related to the external context, which includes the sociopolitical climate, inter-organizational environment, and limited advocacy for increasing service availability. Studies from high-income countries examining the delivery of substance use interventions have similarly identified resource limitations [49], provider knowledge and capacity [50], and inter-organizational coordination [30, 51] as barriers to implementation. The post-conflict context, however, presents unique challenges due to instability, disruptions to pre-existing systems, displacement and accelerated attrition of human resources, and rapid turnover in government and institutional leadership, among other factors. Furthermore, post-conflict settings are heterogeneous. In our review, the majority of studies enrolled refugees who were currently residing in refugee camps, lived among host communities, or had returned to their country of origin. The services afforded to these variable groups may differ based on their context and location, which introduces added variability into the outer context that may impact implementation.

Few studies provided recommendations for strategies to overcome the implementation challenges they experienced. Previous research on stigma reduction, capacity building, and inter-organizational coordination may provide insight into promising strategies for testing in future implementation studies evaluating substance use services in conflict-affected populations. Stigma is a critical barrier that exists across the inner and outer contexts. Skills-based interventions for individuals with substance use disorder appear to be promising strategies for reducing self-stigma for populations in high-income countries [35], but we could not find information on the effectiveness of skills-based stigma reduction interventions for populations using substances in low- and middle-income countries or conflict-affected populations. Task shifting and incorporating peers into intervention delivery may also serve to reduce institutional and provider-related stigma, as has been suggested by research in high-income settings [52]. Further efforts to develop and evaluate contextually appropriate stigma reduction interventions are needed to reduce barriers to accessing or seeking care for substance use disorder in conflict-affected populations. As was done in one study included in this review [27], investing in advocacy efforts with a variety of stakeholders (e.g., community members, law enforcement personnel, policymakers) during the planning phase of implementation may assist in reducing structural stigma, improving inter-organizational coordination, and preventing undue barriers to accessing services. Integration of substance misuse treatment services into existing health systems, such as primary care or mental health and psychosocial support, may also reduce structural stigma and barriers at multiple levels and increase the scope of services available to clients. Furthermore, this strategy aligns with existing recommendations for substance misuse and mental health care in low-resource and humanitarian settings [44, 53]. An advantage of training providers operating within existing systems is that in situations where concerns about the consequences of substance misuse may be heightened (e.g., refugee settings), providing substance misuse services within pre-existing systems and a neutral context may also be more ethically appropriate and improve client safety. Training providers in the provision of substance misuse treatment and prevention interventions must be coupled with efforts to reduce stigma. A systematic review of substance misuse stigma reduction interventions found that educational and critical reflection-based interventions for representatives of public institutions, including health providers, were effective in reducing structural stigma; however, the effects of education-based interventions on stigma in the general population were mixed [35]. Research on the topic has been conducted in high-income countries but as far as we know not in low- and middle-income countries. It is important that this will be done in the future since cultural and contextual factors contributing to stigma—as well as the feasibility and fit of interventions to reduce stigma—may vary significantly.

Recommended interventions provided by lay or specialized providers must be feasible, relevant, and acceptable in the local context. Identifying and restoring services that were available prior to the conflict or displacement may be an appropriate first step in providing services that are acceptable to the local population [8]. In situations where no services have been available historically, dedicating time to understanding the local context and working in partnership with local stakeholders to identify acceptable and relevant intervention strategies and organizational partnerships is critical. To maximize feasibility, it is also imperative that cost-effective interventions are prioritized given likely resource limitations.

When interpreting the results of this review, there are important limitations of that must be considered. First, very few studies were identified in this broad, scoping review. This reflects the lack of research on substance use interventions in conflict-affected populations. This lack of literature limited our ability to make strong conclusions based on the included studies that varied by setting, population, refugee status, intervention modality, evaluation method, etc. Given this heterogeneity, the findings of this review should not replace efforts by practitioners and researchers to thoroughly explore cultural and contextual factors specific to a population and setting that may uniquely impact implementation [54]. An additional limitation relates to the lack of current knowledge on the effectiveness of the interventions included in this review in conflict-affected populations. Most of the included studies did not include rigorous evaluations or detailed descriptions of the interventions. Future operational research should consider hybrid effectiveness-implementation designs that allows for both process and impact outcomes to be explored [55]. It is possible that interventions were not included because we limited our studies to those published in English, and due to systematic exclusion of studies (i.e., publication bias), we may not have access to all relevant reports or articles. Despite these limitations, this study documents the first effort to synthesize literature on the implementation of substance use interventions in conflict-affected populations.

## Conclusions and implications

Although recent studies have found that it is feasible to introduce substance misuse treatment and prevention services in conflict- and post-conflict settings, research on overcoming implementation barriers is still in its infancy. Further operational research is needed to evaluate effective models for service delivery and how to integrate these services into existing systems and to assess the impact of service delivery on substance use and related outcomes. Opportunities for addressing implementation challenges include identifying effective stigma reduction interventions, increasing provider capacity to provide substance misuse services to conflict-affected populations, integrating substance misuse services into existing systems, and generating high-quality data that can be used to advocate for greater prioritization of substance misuse services in policymaking and resource allocation.

## Abbreviations

ASSIST: Alcohol, Smoking and Substance Involvement Screening Test
AUDIT: Alcohol Use Disorders Identification Test
CETA: Common Elements Treatment Approach
EPIS: Exploration, Preparation, Implementation and Sustainment
LINC: Linking Human Systems Community Resilience Model
RCT: Randomized controlled trial
UNODC: United Nations Office on Drugs and Crime
